# A Computational Framework for Tracing the Origins of Genomic Islands in Prokaryotes

**DOI:** 10.1155/2014/732857

**Published:** 2014-10-28

**Authors:** Peng Wan, Dongsheng Che

**Affiliations:** Department of Computer Science, East Stroudsburg University, 200 Prospect Street, East Stroudsburg, PA 18301, USA

## Abstract

Genomic islands (GIs) are chunks of genomic fragments that are acquired from nongenealogical organisms through horizontal gene transfer (HGT). Current researches on studying donor-recipient relationships for HGT are limited at a gene level. As more GIs have been identified and verified, the way of studying donor-recipient relationships can be better modeled by using GIs rather than individual genes. In this paper, we report the development of a computational framework for detecting origins of GIs. The main idea of our computational framework is to identify GIs in a query genome, search candidate genomes that contain genomic regions similar to those GIs in the query genome by BLAST search, and then filter out some candidate genomes if those similar genomic regions are also alien (detected by GI detection tools). We have applied our framework in finding the GI origins for *Mycobacterium tuberculosis* H37Rv, *Herminiimonas arsenicoxydans*, and three *Thermoanaerobacter* species. The predicted results were used to establish the donor-recipient network relationships and visualized by Gephi. Our studies have shown that donor genomes detected by our computational approach were mainly consistent with previous studies. Our framework was implemented with Perl and executed on Windows operating system.

## 1. Introduction

Genomic islands (GIs) are clusters of foreign genes acquired from nongenealogical organisms through horizontal gene transfer (HGT). Previous studies have elucidated some HGT mechanisms, including (i) transformation [1], (ii) conjugation [2], (iii) transduction [3], and (iv) gene transfer agent [4]. The occurrences of HGT make considerable contribution to genome evolution, which is especially evident in pathogenic organisms [5] or antibiotic resistant organisms [6, 7]. Such quantum-leap evolution can cause drastic changes to species, especially in bacteria, and shape the world where humans live [8–10]. Therefore, it is practically significant to explore origins of GIs (i.e., donors of GIs). Besides, the occurrence of HGT between highly divergent organisms has great impact on phylogenetics, which can disclose the evolutionary relationship of organisms. The acquired GIs or biological features may contaminate the correctness of phylogenetic realtions. Therefore, exploring the origin donor of GIs can also contribute to the analysis of genealogical phylogenetics.

Some previous research works of constructing donor-recipient network based on individual genes have been carried out [11, 12], where the network is composed of a set of vertices (i.e., organisms) and edges, which connect vertices and reveal the occurrences of HGT between organisms. By studying the visualized networks, the researchers could uncover the hidden mechanisms of HGT such as genomic biases or barriers [13], DNA repair bypasses [12], and eukaryotes origins and evolutions [11]. While using individual genes for studying donor-recipient network can reveal the mechanisms of HGT to some degree, a HGT event usually occurs in a cluster of sequential genes instead of single genes separately. Therefore, it may not truly reflect the donor-recipient networks accurately if using single-gene-based analysis.

Available GI detection tools these days make it possible to generate donor-recipient network based on GIs. Current GI prediction tools can be grouped into three categories: (1) sequence composition based approach, (2) comparative genome analysis approach, and (3) integrative approach. The sequence composition approach aims to identify some special signatures in the query genome. The most representative signatures include G + C content, dinucleotide biases, codon usage, mobility genes (including transposases and integrases), and tRNA genes [14, 15]. Related tools using this kind of approach include AlienHunter [16], Centroid [17], GIDetector [18], GIHunter [19], PAI IDA [20], and SIGI-HMM [21]. Comparative genome approach uses the phylogenetically close species as reference genomes and identifies subsequences that are unique in the query genomes and treats them to be GIs. The implemented programs of this approach include IslandPick [22] and MobilomeFINDER [23]. The integrative approach uses the prediction results of the above programs to obtain census GIs. Tools of using such approaches include EGID [24] and IslandViewer [25]. The availability of these GI tools, thus, provides us with a chance to study donor-recipient relationship through GIs.

In this paper, we propose using GIs to trace the origins of GIs and study donor-recipient networks across organisms. To make our donor-recipient networks meaningful, we model edges as weighted, which can be represented by the number of GIs that a donor genome contributes to a recipient genome. In a previous study, it was hypothesized that if a HGT happened between two organisms, a series of subsequent directional transfers should be very likely to arise [26]. Therefore, it is reasonable to hypothesize that if two nongenealogical organisms harbor more occurrences of HGT, the more confident donor-recipient relationship is between them.

In this study, we would also like to check whether donor-recipient relationship is random or biased. Previous studies have shown that HGT is not a random event but of preferences to occur [13]. It has been found that HGT occurs among organisms which share similar factors such as genome size, genome G/C composition, carbon utilization, and oxygen tolerance [27]. The paper is organized as follows. In Section 2, we will describe the dataset and our computational framework in detail (it is freely available for noncommercial use at http://www5.esu.edu/cpsc/bioinfo/software/GIDonor). In Section 3, we will illustrate the usage of computational framework on three case studies and discuss our predicted results with visualized networks. Finally, we draw a conclusion in Section 4 and discuss our possible future work.

## 2. Materials and Methods

### 2.1. Data Acquisition

GenBank [28], an open-access resource which is established and maintained by the National Center of Biotechnology Information (NCBI), houses all publicly available biological sequences such as genomic sequences and protein sequences. To trace GI donors, we collected all available prokaryotic nucleotide sequences from GenBank, which covered 162 million sequences with 150 billion nucleotides bases in total.

GenBank provides different data formats for feeding different programs. For local based BLAST [29] search in our framework, a preformatted format was used. This format is small in terms of size, so that it is convenient to download, and it can also be transformed into FASTA format. For AlienHunter [16], used for GI detection in our framework, we used FASTA format. More specifically, we fed AlienHunter with FASTA nucleic acid files with the extension of “.fna.”

### 2.2. Computational Framework

Essentially, our framework relies on two kinds of bioinformatics tools, sequence similarity search tool and GI prediction tool. We used BLAST as the sequence similarity tool since it is accurate and efficient in terms of computing time. We used sequence composition based GI tools for predicting GIs in our framework because this kind of approach can be applied to any query genome sequence.

**Figure 1:**
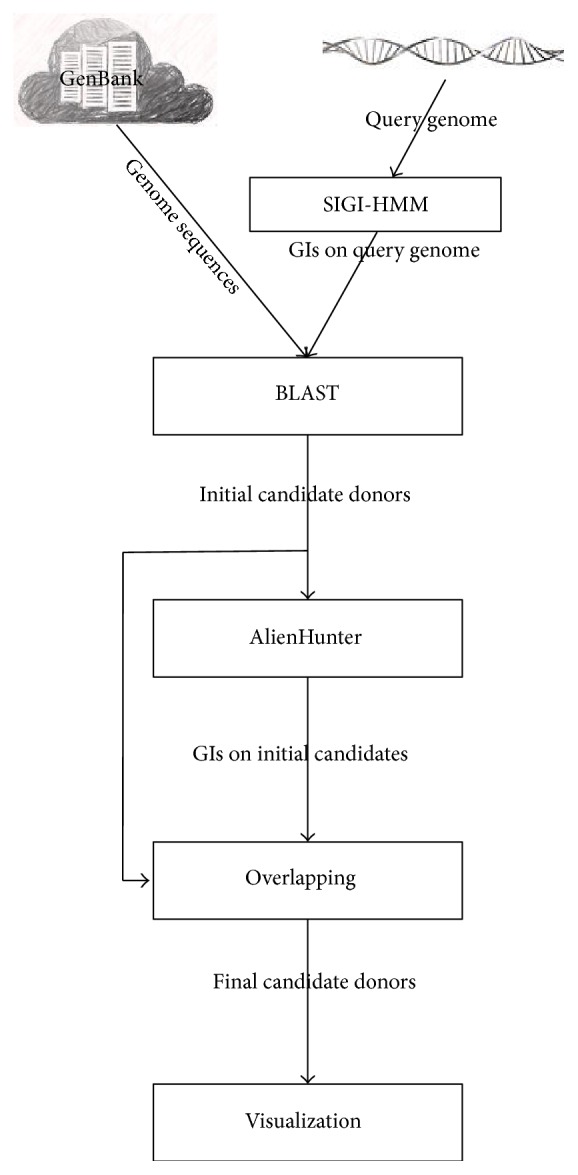
A schematic view of our computational framework for predicting the origins of GIs.

By integrating multiple bioinformatics tools, our framework can process sequenced genomic data automatically and obtain final predicted donor organisms. The framework consists of five steps (also shown in Figure 1), including

- obtaining GIs in a query genome with SIGI-HMM;
- collecting initial candidate donor genomes by BLAST;
- predicting complete GI lists in candidate donor genomes using AlienHunter;
- obtaining final donor genomes through overlapping;
- visualizing donor-recipient relationships.

Each step of our computational framework will be described in the following subsections.

#### 2.2.1. Obtaining GIs in a Query Genome with SIGI-HMM

SIGI-HMM [21], an open-source program, is based on genome composition analysis and is available for local installation, as well as web GUI on IslandViewer [25]. SIGI-HMM first analyzes the codon usage of each gene, provides the score for each gene based on the codon usage, and thus can find alien genes based on codon usage scores. Because SIGI-HMM produces the highest precision rate, guaranteeing the predicted GIs to be true GIs [22], we adopted it as the GI prediction tool in query genomes.

Because IslandViewer has provided predicted GIs by the program of SIGI-HMM, we took advantage of it and obtained predicted GIs for any query genome from IslandViewer. In order to automatically collect GIs provided by IslandViewer, we developed a Perl script to send out query genome information to IslandViewer's server and extract GI ranges from the IslandViewer server.

#### 2.2.2. Collecting Initial Candidate Donor Genomes by BLAST

BLAST is extensively used in sequence similarity measurement due to its efficient computation, high accuracy, and the availability of cross-platforms. Therefore, BLAST was incorporated into the framework for searching initial candidate donor genomes, which should contain genomic fragments similar to GI sequence in the query genomes. In BLAST, the cutoff value *e* is used to evaluate the similarity between genome sequences. In this study, we set *e* value of 10^−20^ as the cutoff to find our candidate donors.

BLAST itself is a suite of multiple programs. In this study, we used* blastn* for nucleotide sequence search. The BLAST output contains information of genome sequence alignments, alignment score, and *e* values, and such information can be processed systematically through open source of Bioperl [30]. Therefore, we wrote a Perl script that uses Bioperl modules to extract relevant information from BLAST outputs.

#### 2.2.3. Predicting Complete GI Lists in Candidate Donor Genomes Using AlienHunter

The initial candidate donor genomes obtained by BLAST search may contain some false positive donors (i.e., the genome fragments similar to those GIs in the query genome are also transferred from donor genomes); thus a filtering process should be conducted to remove such false positives and obtain final donor genomes. Theoretically, the final candidates should possess a characteristic; that is, the detected similar genomic fragments from BLAST should be original (in other words, they are not GIs) in the corresponding hosts. In order to check whether such genomic fragments are original or not, we must use GI tools to check whether they are GIs or not. We incorporated a GI tool AlienHunter [16] in our framework to extract a complete list of GIs for all initial candidate donor genomes.

We chose the AlienHunter tool in this step because previous assessment study on available GI tools [22] had shown that AlienHunter possesses the highest recall rate, compared with other sequence compositions including SIGI-HMM, IslandPath [31], PAI IDA [20], and Centroid [17]. In other words, AlienHunter can predict the most complete lists of GIs in any candidate donor genome. This is very crucial because we can filter out more initial candidate donors and guarantee the final identified donors to true donors. In that sense, our prediction framework for finding donors of GIs is very conservative.

#### 2.2.4. Obtaining Final Donor Genomes through Overlapping

Final donor genomes were obtained by filtering out initial candidate genomes (obtained from Section 2.2.2), where genomic fragments similar to recipients of GIs were also predicted as GIs from Section 2.2.3.

**Figure 2:**
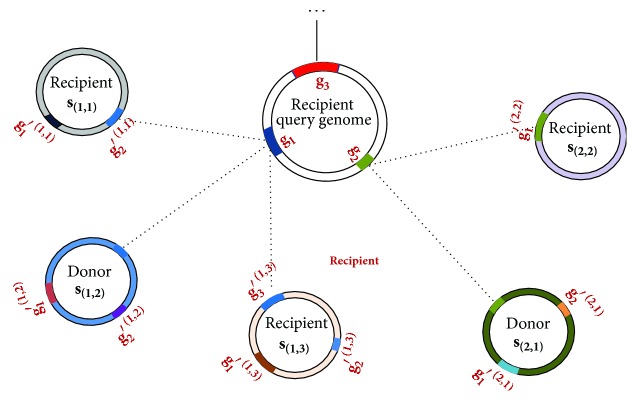
A schematic illustration of overlapping results. The circles represent genomes, with the top middle one denoting a query genome and the rest ones denoting initial candidate donor genomes. The GI set in query genome, *G*, includes {**g**_1_, **g**_2_, **g**_3_}, the initial candidate donor genomes' set, *S*, includes {**s**_(1,1)_, **s**_(1,2)_, **s**_(1,3)_, **s**_(2,1)_, **s**_(2,2)_}, and the detected GIs' set in intial candidate donor genomes, *G*′, includes {**g**_1_′^(1,1)^, **g**_2_′^(1,1)^, **g**_1_′^(1,2)^, **g**_2_′^(1,2)^, **g**_1_′^(1,3)^, **g**_2_′^(1,3)^, **g**_3_′^(1,3)^, **g**_1_′^(2,1)^, **g**_2_′^(2,1)^, **g**_1_′^(2,2)^}. Only those candidate donor genomes whose similar subsequences are not GIs are predicted to be donors. Otherwise, the candidates are predicted as recipients. For instance, the initial candidate donor **s**_(1,1)_ is labeled as recipient because **g**_1_ is similar to **g**_2_′^(1,1)^, a member of GI set, *G*′, of **s**_(1,1)_. The initial candidate donor **s**_(1,2)_ is labeled as donor because the **g**_1_'s similar subsequence (the sequence was not labeled but was highlighed and connected to **g**_1_ with a dashed line) does not overlap with any member in the GI set, *G*′, of **s**_(1,2)_. In this example, **s**_(1,2)_ and **s**_(2,1)_ are predicted to be final donor genomes for the query genome.

More formally, let a set ∑{*G*, *S*, *G*′} represent the information from previous steps, where *G* denotes the set of GIs of a query genome obtained from SIGI-HMM, *S* denotes the set of initial candidate donor genomes obtained from BLAST, and *G*′ denotes the set of GIs on initial candidate genomes predicted from AlienHunter. Furthermore, for each of them,

(i) *G* = {*g*_*i*_∣*i* ≥ 0},
(ii) *S* = {*s*_(*i*,*j*)_∣*i*, *j* ≥ 0},
(iii) *G*′ = {*g*_*μ*_′^(*i*,*j*)^∣*i*, *j*, *μ* ≥ 0},

where *g*_*i*_ denotes the *i*th GI in query genome, *s*_(*i*,*j*)_ denotes the *j*th initial candidate donor for potentially donating *i*th GI (*g*_*i*_) on query genome, and *g*_*μ*_′^(*i*,*j*)^ denotes *u*th GI for a candidate donor (*s*_(*i*,*j*)_). The overlapping operation is illustrated in Figure 2 in detail. This process was implemented in Perl script.

#### 2.2.5. Visualizing Donor-Recipient Relationships

For any given query genome, a list of final candidate donor genomes can be obtained by running the previous steps in our framework. In order to quantitatively visualize the number of donations from donors, we intended to produce a donor-recipient weighted network, where nodes denote donors and the recipient and the weights corresponding to edges are the number of HGT occurrences (i.e., quantified by the GI transfers) between two organisms. By looking at heavy weighted edges in networks, we can easily recommend highly potential donors.

Gephi [32] is an open-source tool for network analysis and visualization. The network from Gephi can be either directed or undirected. It was implemented with JAVA, and hence it is platform-independent. Therefore, we utilized it to generate the donor-recipient network in our framework.

## 3. Results

We have applied our computational framework on three bacterial cases, including donor prediction of* Mycobacterium tuberculosis* H37Rv,* Herminiimonas arsenicoxydans*, and a HGT network establishment of three species in* Thermoanaerobacter* (*T. pseudethanolicus* ATCC 33223,* T. tengcongensis*, and *T.* strain X514). The possible HGT mechanisms of these species had been studied previously, and thus our predicted results could be investigated by comparing previous studies.

### 3.1. Predicted Donors of* Mycobacterium tuberculosis* H37Rv

*Mycobacterium tuberculosis* (*M. tuberculosis*) is a bacterial species that causes most cases of tuberculosis (TB) [33].* M. tuberculosis* H37Rv is the best characterized strain of* M. tuberculosis* and had been sequenced and analyzed biologically [34]. Previous work that combined genome-wide parametric analysis showed that* M. tuberculosis* encompassed 48 GIs, which consist of 4.5% of the genome (199 kb), and include 256 genes [35, 36]. Through applying our framework in* M. tuberculosis* H37Rv, we hope to reveal the origins of GIs and provide a complete list of GI donors to substantially enrich the information of mycobacterial pathogenicity.

**Figure 3:**
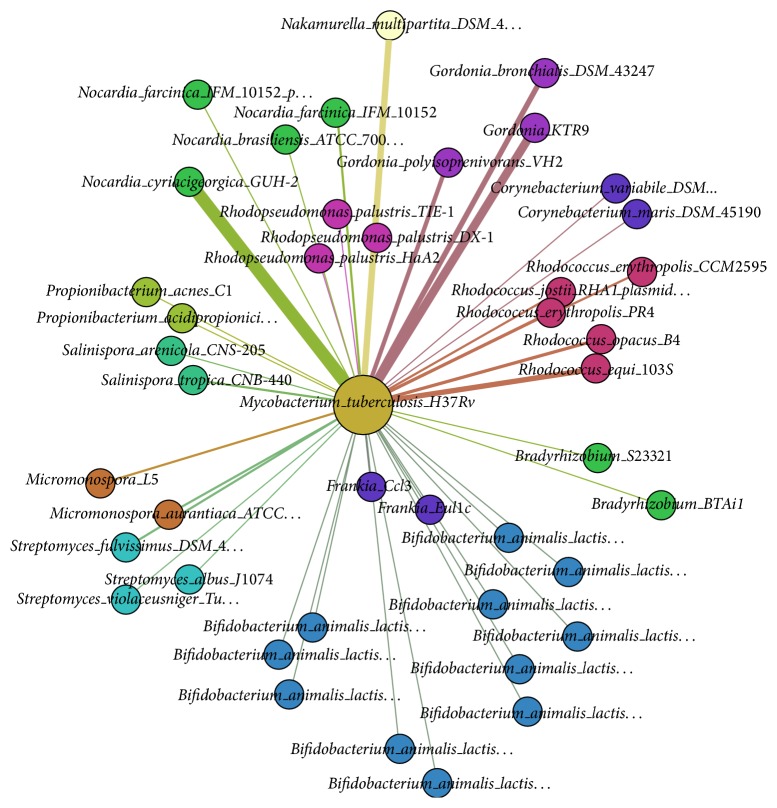
The network of predicted* Mycobacterium tuberculosis* H37Rv's donors. The undirected network demonstrates all candidate donor genomes of* Mycobacterium tuberculosis* H37Rv. The weight of each edge denotes the number of donated GIs. Each color represents one genus of species.

**Figure 4:**
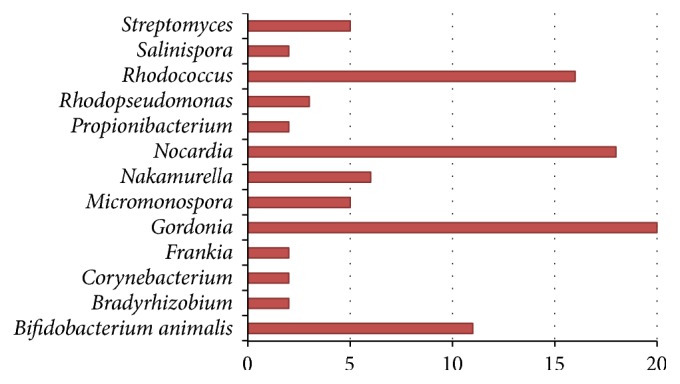
The predicted donors of* Mycobacterium tuberculosis* H37Rv. The candidate donors were grouped as genus, and the number of donated GIs was represented by the length of bars.

Figure 3 displays all predicted final candidate donors from our framework, where species with the same genus have been dyed with the same colors and positioned next to each other for better visualization. As shown in Figure 3, the predicted donor genus which contributed the most is* Gordonia*, followed by* Nocardia* and* Rhodococcus* in descending rank. The network met the power law degree. The number of GIs, in which each genus of donors contributed to the recipient H37Rv, has been summarized in Figure 4.

When comparing our predicted donors of H37Rv's GIs with the predicted potential donors from previous studies [35], we found that there are a lot of matching predictions. For instance, three donors with the highest donations, including* Gordonia*,* Nocardia*, and* Rhodococcus*, were also detected as their potential origins of GIs (Figures 3 and 4). Besides, we also incorporated* Bradyrhizobium* and* Corynebacterium* into our final candidate donors. All of those were also present in previous studies. In addition, our framework has detected a new potential donor,* Bifidobacterium animalis*, which has not been reported in previous studies. We strongly believe in this as one of the high potential donors since* B. animalis* resides in the body of most mammals including humans, which may interact with* M. tuberculosis* and transfer genes to* M. tuberculosis*.

### 3.2. Predicted Donors of* Herminiimonas arsenicoxydans*

*Herminiimonas arsenicoxydans* (*H. arsenicoxydans*) is an arsenite-oxidizing bacterium that belongs to heterotrophic Betaproteobacterium [37]. It was isolated from heavy-contaminated sludge with arsenic and other heavy metals from industrial wastewater treatment plants. Under aerobic conditions, it can oxidize arsenite to arsenate and produce at least two arsenate reductases. It was revealed to be able to resist against numerous heavy metals such as Se, Mn, Cr, Cd, Sb, and Ni. All of those competences enable its widely ecological usage in arsenical polluted environment [37]. A better understanding of* H. arsenicoxydans* can be beneficial for arsenical polluted remediation.

**Figure 5:**
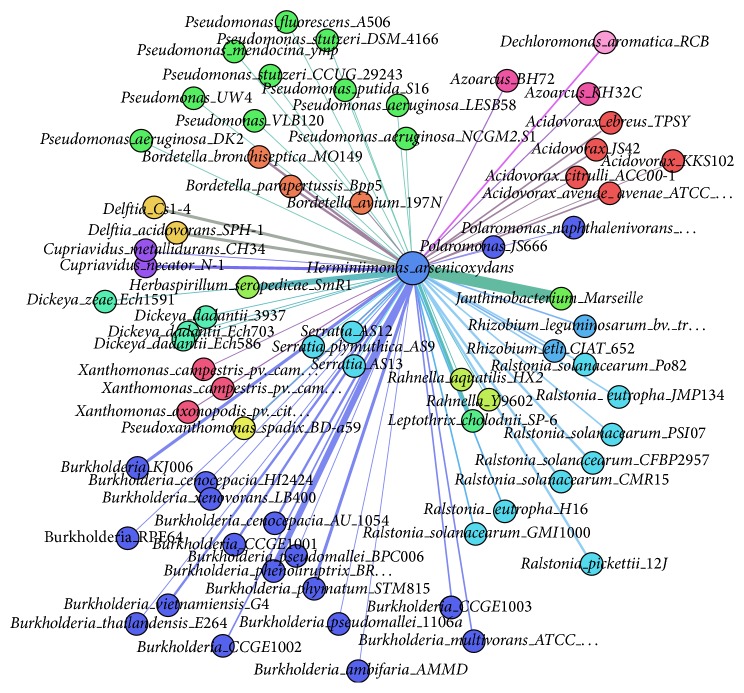
The network of predicted* Herminiimonas arsenicoxydans*' donors. The undirected network demonstrates all candidate donor genomes of* Herminiimonas arsenicoxydans*. The weight of each edge denotes the number of donated GIs. Each color represents one genus of species.

**Figure 6:**
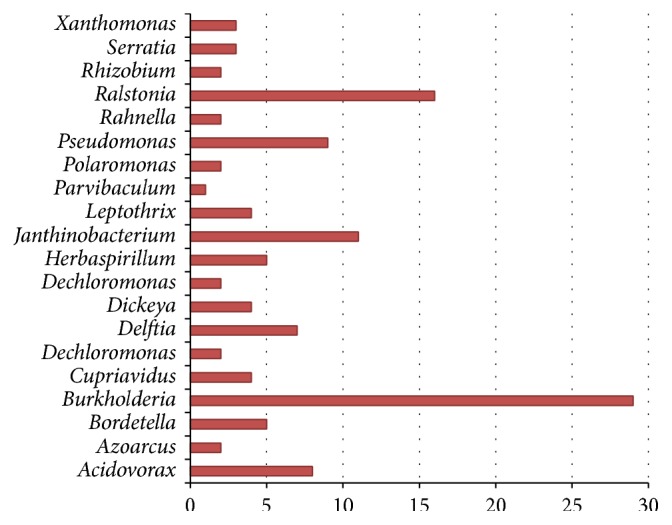
The predicted donors of* Herminiimonas arsenicoxydans*. The candidate donors were grouped as genus, and the number of donated GIs was represented by the length of bars.

Our predicted donors of* H. arsenicoxydans* are shown in Figures 5 and 6. Like previous network, species of the same genus have the same colors, and they are placed next to each other in the network. As we can see in the network of Figure 5,* Janthinobacterium *Marseille (*J. *Marseille) is a highly frequent donor (the occurrence of HGTs is 11 times). Actually,* J. *Marseille belongs to the order Burkholderiales, which has been reported to be the major gene donation source for* H. arsenicoxydans* [12]. Both our predicted results and previous studies strongly indicate that this is the donor for* H. arsenicoxydans*. We also notice that the number contributed by the genus of* Ralstonia* is 16 times, which is also very significant. These findings strongly indicate that species in the Burkholderiales order are major contributors to GIs found in* H. arsenicoxydans*. Another noticeable GIs contributor was from the Pseudomonadales order (Figures 5 and 6), which was also consistent with previous study [12]. Therefore, we can conclude that the origins of GIs in the genome of* H. arsenicoxydans* are from Burkholderiales and Pseudomonadales.

### 3.3. Predicted Donors of* Thermoanaerobacter*

*Thermoanaerobacter* is a genus that contains a cluster of thermophilic and anaerobic bacteria distributed in high-temperature surroundings like springs. They can absorb heat from surroundings as their source of energy to drive their metabolism [38]. Therefore, it will be particularly interesting to study the adaptability of thermophile, examine the evolution path, and ultimately uncover the origins of such special features.

**Figure 7:**
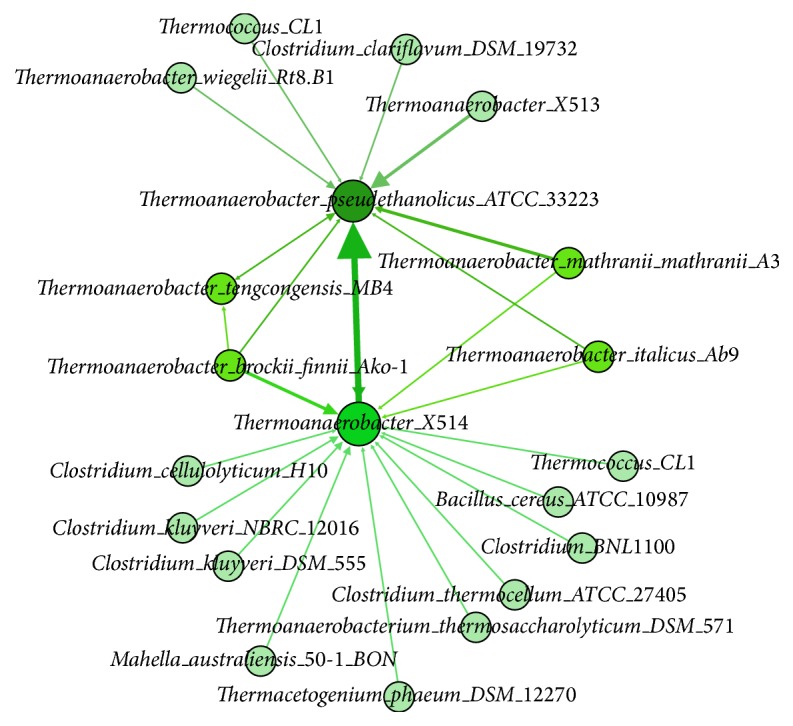
A directed network of donor-recipient relationship based on* T. pseudethanolicus *strain ATCC33223,* T. tengcongensis* MB4, and *T*. strain X514. The directed network demonstrates the GI's transfer direction. The weight of each edge denotes the number of occurrences of HGT.

**Figure 8:**
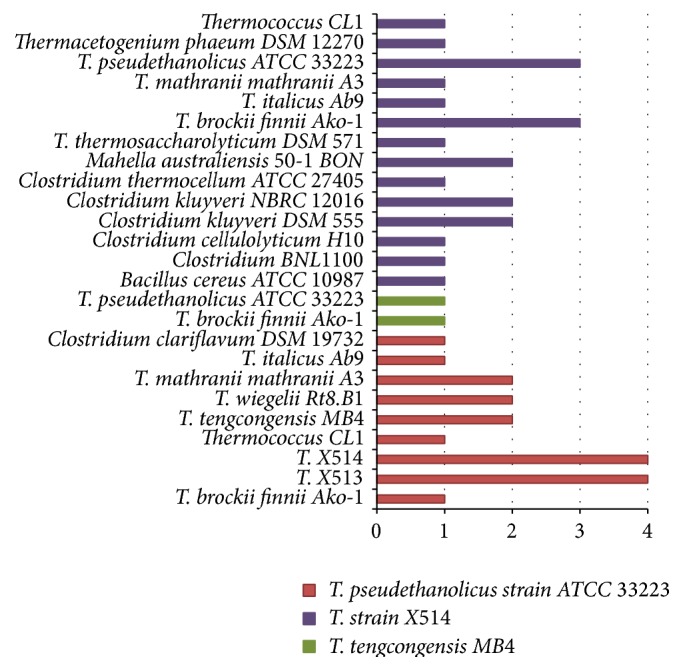
The predicted donors of* Thermoanaerobacter*. The chart consists of three recipients which have been labeled with different colorful bars. Each bar represents the number of donated GIs from one specific donor for a corresponding recipient.

A simplified directed network of donor-recipient relationship has been generated in previous work [12]. The directed network was focused on three members of* Thermoanaerobacter*, including* T. pseudethanolicus *strain ATCC33223,* T. tengcongensis,* and* T*. strain X514. We extended the previous network with more predicted donors as shown in Figures 7 and 8. As you can see, our directed network has expanded previously studied network on a basically consistent condition [12]. For instance, we predicted one GI in* T*. strain X514 that originally belongs to* Clostridium thermocellum* ATCC 27405. This is consistent with their predicted result even though they considered it as a mediator, which played an agent role in the transfer. We also detected seven GI transfers between* T*.* pseudethanolicus *strain ATCC33223 and* T*. strain X514 and one GI transfer from* T*.* pseudethanolicus* strain ATCC33223 to* T. tengcongensis* MB4. Most noticeably,* T. brockii finnii* Ako-1 was present as the donor for all three query genomes as shown in Figure 5. We hypothesize that* T. brockii finnii* Ako-1 interacts with these three* Thermoanaerobacter* species and may contribute heat-resistant related genes to these species.

## 4. Conclusions and Discussion

HGT has provided organisms apportunities to obtain special features from foreign organisms. In order to reveal genetic sources of HGT, we have developed a computational framework for identifying origins of HGT through predicted GIs. The predicted results in this study were consistent with previous studies, strongly indicating the correctness of our computational framework overall. In addition, our framework has also discovered some new donors not reported in previous studies, and further investigation of these new discoved donors indicates that they are possible donors in the biological and evolutionary perspective.

While there is no standard benchmark to measure the accuracy of our framework, it should be noted that the accuracy of our framework depends on the performance of other prediction tools. On the one hand, the selection of cutoff value used in BLAST will affect our prediction results. On the other hand, we adopted sequence composition analysis tools, which is generally applicable to any sequenced query genome, but the prediction is not the most accurate one when compared to comparative genome analysis GI tools. Furthermore, the computation time for finding GIs for a genome is very expensive. For instance, a genome with a typic genome size will take about one hour to complete the entire process of GI search. Thus, for a query genome, it could generate dozens of initial candidate donor genomes, and thus it takes about a day and night to complete the whole process.

In this study, we just studied three query genomes, for the purpose of testing the correctness of our computational framework. In the next stage, we will use a cluster of computers or superpower computers, to predict HGT donors for all sequenced bacterial and archaea genomes using our framework. We will build a large-scale donor-recipient network based on all the results. We believe that such kind of networks will reveal a panoramic view of gene transfer information across microbial organisms and provide some insights into evolutionary biologists to study genome evolution.
